# Point-of-Care Compatibility of Ultra-Sensitive Detection Techniques for the Cardiac Biomarker Troponin I—Challenges and Potential Value

**DOI:** 10.3390/bios8040114

**Published:** 2018-11-21

**Authors:** Brian Regan, Richard O’Kennedy, David Collins

**Affiliations:** 1 School of Biotechnology, Dublin City University, 9 Dublin, Ireland; richard.okennedy@dcu.ie (R.O.); david.collins@dcu.ie (D.C.); 2 Research Complex, Hamad Bin Khalifa University, Qatar Foundation, P.O. Box 34110 Doha, Qatar

**Keywords:** point-of-care, cardiac biomarkers, ultra-sensitive assays, detection mechanisms, nanomaterials

## Abstract

Cardiac biomarkers are frequently measured to provide guidance on the well-being of a patient in relation to cardiac health with many assays having been developed and widely utilised in clinical assessment. Effectively treating and managing cardiovascular disease (CVD) relies on swiftly responding to signs of cardiac symptoms, thus providing a basis for enhanced patient management and an overall better health outcome. Ultra-sensitive cardiac biomarker detection techniques play a pivotal role in improving the diagnostic capacity of an assay and thus enabling a better-informed decision. However, currently, the typical approach taken within healthcare depends on centralised laboratories performing analysis of cardiac biomarkers, thus restricting the roll-out of rapid diagnostics. Point-of-care testing (POCT) involves conducting the diagnostic test in the presence of the patient, with a short turnaround time, requiring small sample volumes without compromising the sensitivity of the assay. This technology is ideal for combatting CVD, thus the formulation of ultra-sensitive assays and the design of biosensors will be critically evaluated, focusing on the feasibility of these techniques for point-of-care (POC) integration. Moreover, there are several key factors, which in combination, contribute to the development of ultra-sensitive techniques, namely the incorporation of nanomaterials for sensitivity enhancement and manipulation of labelling methods. This review will explore the latest developments in cardiac biomarker detection, primarily focusing on the detection of cardiac troponin I (cTnI). Highly sensitive detection of cTnI is of paramount importance regarding the rapid rule-in/rule-out of acute myocardial infarction (AMI). Thus the challenges encountered during cTnI measurements are outlined in detail to assist in demonstrating the drawbacks of current commercial assays and the obstructions to standardisation. Furthermore, the added benefits of introducing multi-biomarker panels are reviewed, several key biomarkers are evaluated and the analytical benefits provided by multimarkers-based methods are highlighted.

## 1. Introduction

Point-of-care testing (POCT) is becoming an increasingly critical diagnostic approach given the need for rapid turnaround of results, low sample consumption and its potential to offer high sensitivity detection in a user-friendly manner. POCT ultimately represents a test in which the result will contribute to a strategic decision, leading to improved patient management and an overall better health outcome [1]. It is an ideal approach to implement for express medical screening, with the potential to considerably alleviate emergency department (ED) waiting times. POCT can be conducted in virtually any setting with an ever-expanding variety of devices commercially available, ranging from basic handheld test strips to complex microfluidics-based benchtop instruments. Hence, a potential consequence of POCT is the decentralisation of aspects of healthcare, leading to a reduction in overall hospital and laboratory facility costs, in turn offsetting the average increase in cost per test [1]. This technology is becoming increasingly influential in how CVD is diagnosed and managed, with a broad range of biochemical markers being used to assist the detection and subsequent treatment of cardiac-related illnesses.

Numerous cardiac biomarkers have been identified, with some being more specific than others and offering varying diagnostic and prognostic capabilities [2,3,4]. Importantly, biomarkers should provide crucial information on the health status of a patient, while assisting in patient management and risk stratification [5]. The cardiac troponins (cTn) are among the most utilised cardiac biomarkers, with cardiac troponin I (cTnI), a protein found only in the myocardium, often being considered as the gold standard for cardiac biomarkers, with the European Society of Cardiology (ESC) recommending its use to rule-in/rule-out acute myocardial infarction (AMI) [6,7]. Additionally, the ESC has stated that the latest high sensitivity cTn (hs-cTn) assays have enabled a change in the way in which cTn testing is performed, reducing the testing time from 3 to 1 h [7]. This capability is driven by the increasing sensitivities achievable by hs-cTn assays and thus reducing the negative impact of the “troponin blind” period, which is widely considered as one of the most beneficial aspects of the new assays [8]. The troponin blind period is traditionally the initial period during which troponin levels are not detectable using the standard cTn assays though AMI symptoms may be present [9]. Evidently, the latest hs-cTn assays have not only reduced this period, but have also the potential to detect cTn in the 99th percentile with all hs-cTn assays demonstrating the ability to measure cTn in over 50% of the healthy population [10]. These new levels of performance have facilitated faster and more accurate diagnosis for CVD. However, these exceptional achievements are yet to be replicated on POC platforms, which underperform in comparison with particular differences apparent for detection sensitivity and test variability [11,12,13]. 

Ongoing studies dedicated to increasing the sensitivity of cTn assays and biosensors which will be presented throughout this review, with many research groups capitalising on emerging technologies to achieve sensitivities which can potentially detect cTn in the 99th percentile. For a cTn assay to be considered as a high sensitivity assay, it must be capable of detecting cTn in at least 50% of healthy individuals in conjunction with achieving a required level of precision [14]. Taking this a step further, for a cTn assay to be classified as ultra-sensitive, Jarolim states that it should be able to detect cTn in the 99th percentile of healthy individuals [15]. Currently, the lack of standardisation between cTnI assays and the variation of cTnI concentrations among gender- and age-specific groups leads to an unestablished universal upper reference limit (URL) [16,17]. The URL is used to assist in the definition of AMI as cTnI levels exceeding this threshold indicate myocardial damage [18]. Defining the URL requires obtaining serum samples from healthy individuals and using statistical analysis to determine a boundary cTnI level [19,20]. Furthermore, in the case of cTnI, each commercial assay will have a distinct URL defined in pre-clinical tests, adding to the uncertainty surrounding the development of a universal URL. Hence, it is not plausible to assign a rigid cTnI concentration by which to evaluate the reviewed assays and biosensors, and until clinical trials have been conducted, the full extent to which an assay or biosensor can consistently detect cTnI is not truly known. 

There are a variety of methodologies and detection stratagems that have been developed for cTnI detection, ranging from electrochemical to luminescence and optical to FET-based, with a vast diversity of detection mechanisms having been reported [21,22,23]. These different approaches have associated implications affecting their point-of-care (POC) compatibility. Numerous methods sometimes require expensive and complex instrumentation, and sophisticated bioconjugation strategies for immobilization and/or Ab labelling, potentially restricting integration into POC platforms. Others, however, can be easily miniaturised and made portable, may not require Ab labels and can be fabricated at low cost. Such inherent characteristics will increase the appeal of certain techniques and reduce that of others in relation to the fabrication of a POC platform. However, the design of a POC platform will be heavily influenced by a set of distinctive criteria outlined prior to fabrication and can be specific to each individual health condition. Evaluating the various detection techniques in regard to POC compatibility requires briefly considering the practical challenges associated with each approach and as crucial as the real-world challenges of POC fabrication are in relation to distinguishing between suitable detection mechanisms, However, the primary objective is the potential of each individual technique to detect cTnI at ultra-sensitive levels whilst adhering to POC standards requirements.

The aim of this review is to assess the latest research developments involving cTnI immunoassays and biosensors from 2015 until the present, with a focus on evaluating their suitability towards integration into POC platforms. Considerable attention is focused on the detection method employed, as this is often considered asone of the most fundamental elements of a POC platform. The methods of immobilising recognition elements and the use of nanostructures will be reviewed in an attempt to illustrate the most compatible approaches for the development of highly sensitive POC platforms. Furthermore, the aggregating factors which potentially contribute towards the development of a POC platform with the capacity to provide effective prognostic and diagnostic capabilities will be discussed. In addition, identification of the beneficial aspects associated with the use of multiplexing for enhanced detection of disease will be examined.

## 2. Complexity of cTnI Detection

Despite the advances made regarding sensing technology, which have culminated in a general improvement in assay sensitivity, detecting cTnI with high precision is still particularly challenging. The range of commercially available biorecognition elements for cTnI is continuously expanding and although the biological basis remains somewhat consistent, different bioreceptors have certain benefits or drawbacks that may make them more or less preferable for particular applications. However, when considering a suitable biorecognition element there are some limitations that often apply and this is especially relevant in the case of cTnI. The ESC has stated that the biorecognition element should target epitopes in the stable region of the amino acid sequence between 30 and 110, as upon release into the circulatory system, the troponin complex “undergoes substantial modification” [24,25]. As well as being circulated in its uncomplexed individual form, cTnI can be present in a binary (most often) or ternary complex. Additionally, cTnI has a half-life that can vary significantly depending upon the particular type of infarction or the cardiac medical condition present, potentially fluctuating by over 12 h for certain cases [26,27]. Another crucial factor that impacts assay performance is the presence of autoantibodies to cTn. Studies have shown that autoantibodies to cTnI are generated by some patients following an AMI, which can impact levels detected [28]. Furthermore, several studies indicate that an interfering factor negatively effects the stable region of the protein, impeding Ab binding as a result of its presence and potentially accounting for the under evaluation of cTnI levels [29,30]. They report that Abs against epitopes near the C- and N-termini are substantially less affected by the interfering factor, conflicting the ESCs recommendations. The primary component of the interfering substances was found to be circulating autoantibodies and as cTnI is often present in a binary or ternary complex, autoantibodies against troponin C (TnC) or cardiac troponin T (cTnT) can also impede sufficient capture of cTnI. Another study has shown that the autoantibodies reactive with cTnI are heterogeneous and are likely to bind to any region of the amino acid sequence, thus once more potentially inhibiting the effective capture of the antigen [31]. Other factors that can lead to distorted cTnI measurements, potentially producing false positives and possibly false negatives, are the presence of heterophile antibodies and rheumatoid arthritis [32,33,34]. Thus, the combination of interferences and variances affecting cTnI measurements complicates the production of consistent techniques to accurately detect cTnI and often creates a disparity between measurements of the same approach.

Many Ab manufacturers supply Abs against a diverse range of cTnI epitopes, including those outside of the stable region. Two separate tables published by the International Federation of Clinical Chemistry (IFCC) display the epitopes targeted by the capture and detection Abs of cTnI assays, with almost all manufacturers targeting the stable region [35,36]. For example, a fluorescence-based immunosensor, that utilises polymer linkers to ensure the fluorophores maintain a fixed distance from one another to reduce the negative effects of quenching, has used two different types of antibodies to create a sandwich assay format [37]. The authors have used monoclonal Ab clones 19C7 and 560 which target areas between amino acids 41–49 and 83–93, respectively, targeting epitopes within the stable region of cTnI. Han et al. have developed novel retroreflective Janus microparticles as immunosensing probes in an optical detection-based immunoassay [38]. This work presents a nonspectroscopic optical technique that relies on basic instrumentation for cTnI measurement, hence it is an excellent candidate for POC integration. The group have selected monoclonal Ab (625 clone) to capture the cTnI which targets the region between amino acids 169 and 178. Nonetheless, the immunoassay was capable of achieving a LOD of 50 ng/L. This outcome is reverberated by Savukoski et al. in their development of a novel cTnI assay targeting epitopes at the N- and C-termini as well as the stable region [39]. This study has shown that through utilising capture antibodies for both termini and the mid-fragment stable region, they were able to minimise the impact of circulating autoantibodies on the cTnI measurements.

The range of issues that must be taken into consideration when developing a highly effective assay for cTnI is very considerable, as already outlined, and many of the assays described in the literature may have severe limitations for accurate determinations of clinically relevant levels associated with disease. Taking an assay from laboratory testing using spiked samples to clinical implementation is a major challenge which requires the assay composition to be rigorously examined. Many of the detection techniques that are reviewed here have not considered many of the practical implications and significant assay challenges associated with cTnI measurements and, thus, the means of the detection employed, the analytical performance of the technique and the suitability for POC integration are the principal features that will now be considered.

## 3. Biomarker Recognition

Biosensors are heavily implicated within the shift towards POCT, enabling elements generally associated with an assay to be packaged into a highly compatible automated platform. Biosensors are composed of two primary components, a recognition element and a transducer, with some considering the signal processing system as the third primary component [40,41]. The recognition element captures the target analyte, enabling the isolation of the analyte and thus facilitating quantitative analysis. There is a broad range of recognition elements commonly used to capture target analytes, such as antibodies, affimers, aptamers, molecular recognition polymers, nucleic acids, lectins and enzymes. The selection of a suitable biorecognition element is often dependent upon preference with each type demonstrating certain distinct advantages. 

### 3.1. Immunosensors

In the case of an immunoassay/immunosensor, the recognition element is an antibody (Ab). In relation to Abs, various types (polyclonal, monoclonal and recombinant) are often utilised in immunoassays with each having different characteristics together with different approaches for production. Polyclonal Abs are generated through the immunisation of the host by a particular antigen (targeted biomarkers such as cTnI) resulting in an immune response that produces a polyclonal antiserum containing antibodies to the immunised antigen but also other antibodies present in the host’s serum [42]. These Abs will be produced against several epitopes of the antigen unlike monoclonal Abs which are produced against a single epitope and are derived from a specific B-cell [43]. An antibody, such as IgG, is comprised of two identical heavy and light chains and can be described as having a Y-shape structure, binding to the antigen at the tip of this structure at areas referred to as the complementarity-determining regions. Recombinant antibodies are produced by exploiting genetic techniques to generate a variety of antibody constructs, such as Fab (fragment antigen binding) Abs and scFv (single-chain fragment variable) Abs [44]. The production method used here offers considerable advantages, such as the Ab size being smaller, enabling a greater density of Abs to be immobilised, and the capacity to add tags to enhance immobilisation and ensure correct orientation [45,46]. A study comparing the use of Ab fragments has shown that their incorporation into an assay may reduce the interference from matrix biomolecules, in addition to reducing reagent consumption and increasing the assay sensitivity [47]. 

In relation to cTnI detection, Xu et al. developed an electrochemical immunosensor in which antibodies were used as the recognition element [48]. In this instance, the Abs had been immobilised on palladium(pd) and platinum (pt) nanoparticles. Monoclonal Abs were used as the biorecognition element in a fluorescence-based immunoassay developed by Ham et al. [49]. This assay uses monoclonal capturing and detection Abs in which the detection process is dictated by temperature due to the magnetic/non-magnetic properties of the fluorescently-labelled La_(1−*x*)_Sr*_x_*MnO_3_ nanoparticles. This unique characteristic allows improved control over the detection process, simply through regulation of the system’s temperature. The use of recombinant Abs in recent cTnI assays is not yet widely reported, with the majority of immunoassays employing the use of monoclonal Abs. However, one group have opted to use recombinant Abs for cTnI detection in a luminescence-based immunoassay, citing the reduction in non-specific signals from the sample matrix due to the absence of the Fc region as an influencing factor [50]. This assay can detect cTnI at ng/L concentrations directly in plasma, a feature that substantially increases its compatibility towards POC integration. There are some other examples of recombinant Abs being utilised as the biorecognition element for markers associated with cardiac disease, although these do not incorporate cTnI detection [51,52,53].

### 3.2. Alternative Biorecognition Elements

Several reports in the literature detail alternative biorecognition elements to Abs for the development of cTnI biosensors. Aptamers are quite a popular choice, with some reporting superior aspects such as thermal stability, ease of modification and low-cost production [54,55]. They are single-stranded oligonucleotides with the capability to selectively bind to target molecules possessing similar qualities to Abs, with distinct advantages and disadvantages associated with them for particular applications [56]. Yang et al. employ the use of both Abs and aptamers in the operation of an electrochemiluminescence (ECL) biosensor array [57]. The aptamers were used to capture the cTnI, with the biotinylated Abs conjugated with ruthenium complexes-labelled streptavidin producing an ECL signal for detection. This approach had a LOD of 0.79 ng/L and a linear detection range of 1–10 ng/L. A different ECL assay uses peptides as the recognition element and aptamers as the basis to create a super-sandwich for signal amplification [58]. This is a technique which is gradually becoming more popular, tackling sensitivity issues associated with standard sandwich assays [59]. It involves the hybridisation of numerous DNA strands, creating a DNA chain in which multiple signal probes can intercalate. Here, the aptamer hybridises with the two ss-DNA probes to form a long-range ds-DNA, in which the ECL signal probes have been attached. The authors have shown that the super-sandwich assay produces almost a 5-fold signal amplification in comparison to the standard sandwich approach, resulting in a LOD of 300 pg/L, but a relatively narrow detection range between 800 pg/L and 10 ng/L. In addition to presenting a new technique for signal amplification, this assay has illustrated that peptides are also suitable biorecognition elements. The majority of cTnI detection techniques rely on a biorecognition element to target a specific antigen, however, there are alternative approaches that can be implemented in the capturing of antigens. Molecularly imprinted polymers (MIPs) are recognition elements that have several impressions of target analyte used for binding throughout their structure [60]. They sometimes have the distinct advantage of having greater thermal stability than Abs, longer storage times, can be repeatedly used and can often be produced more cheaply than Abs. Ma et al. have presented an electrochemical sensor, using MIPs to capture cTnI [61]. The outcome of this work produced a highly sensitive MIP-based cTnI electrochemical sensor capable of detecting cTnI as low as 0.8 ng/L. 

## 4. Immobilisation Strategies

A significant factor in assay or biosensor development is the design of a suitable immobilisation strategy. In relation to POCT, Ab immobilisation strategies affect the biosensor storage and operational stability, imposing a ‘knock-on’ effect on the shelf-life of the POC cartridge [62]. Additionally, this process has considerable implications on the sensor performance and requires detailed attention to ensure the detection capability is not restricted [63]. An immobilisation strategy encompasses the means of effectively and securely fixing the biorecognition element to a surface in such a manner as to maximise the surface area and optimally position the bioreceptor. Certain strategies are carefully selected to increase the bioreceptor immobilisation efficiency which is heavily reliant on Ab orientation. Ideally, Abs should be positioned ‘end up’ to maximise the binding capacity for the antigen, however to date, no known strategy can ensure a consistent Ab arrangement. Nonetheless, some approaches are capable of providing highly orientated Abs through specialised site-specific orientation strategies. 

**Figure 1:**
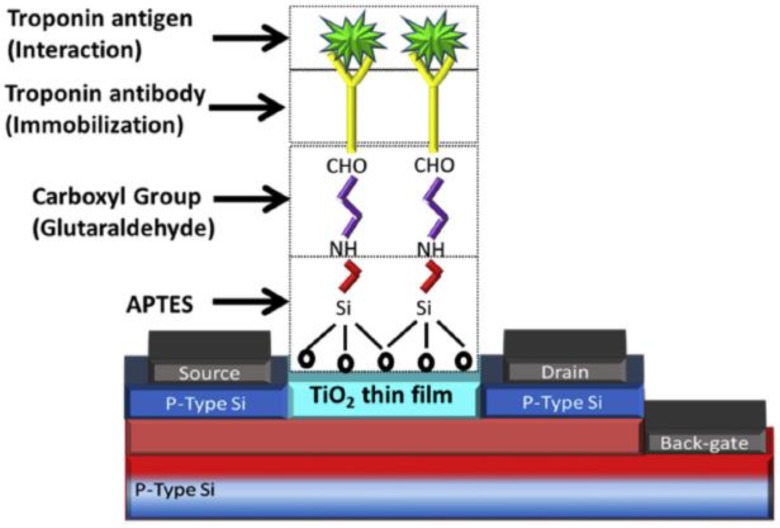
Illustration of FET–based immunosensor depicting the immobilisation strategy [66] (Figure wasre-printed with permission from the publisher).

Physical adsorption is often used and relies on weak attractions such as van der Waals forces, hydrophobic interactions and hydrogen bonding to achieve antibody immobilisation [64]. However, such a strategy results in randomly orientated antibodies on the surface but the amount of antibody capable of binding the antigen may be sufficient to allow the generation of an adequately performing assay. Liu et al. have presented a fibre optic-based biosensor in which Bragg gratings were written into the microfiber to improve the performance of the sensor [65]. The functionalisation of the phase-shifted microfiber Bragg grating probe was achieved through the use of poly-l-lysine, a polyamino acid charged enhancer which generates an attraction between the surface and the Abs. This method is often the basis of immobilisation when performing ELISA using well plates. Forming covalent bonds between Ab functional groups and the substrate seems to be the most popular approach to follow with numerous groups pursuing this method. Adzhri et al. have developed a FET-based immunosensor using 3-Aminopropyltriethoxysilane (APTES) and glutaraldehyde (GA) as the linkers between the TiO_2_ thin film and the cAbs which can detect cTnI as low as 1 μg/L [66]. APTES is often used in the immobilisation strategy for FET biosensors as the chemical nature of the silica layer makes biomolecule immobilisation difficult with standard techniques [67]. Figure 1 provides an indication of the discussed immobilisation chemical structure used in FET biosensors. Similar variations of the same strategy are employed in several other FET biosensors targeting cTnI [68,69,70].

Alternative reagents and crosslinkers are frequently used in biorecognition element immobilisation with a particularly relevant combination of 1-ethyl-3-(3-dimethylaminopropyl) carbodiimide hydrochloride (EDC) and N-hydroxysuccinimide (NHS) regularly employed. The development of an optical microfiber coupler biosensor operating near the turning point region to maximise the refractive index sensitivity involves the use of the EDC/NHS approach to target the Ab amine groups [71]. The EDC activates the carboxyl groups and forms amide crosslinks with the Abs through a hydrolysis reaction which is assisted by the NHS molecules [72]. Another common Ab immobilisation approach is based on the highly specific interaction between streptavidin and biotin [73]. Han et al. have made use of this relationship in the development of a photodiode array biochip by using a NHS-LC-Biotin kit as a means to create biotinylated Abs. The formation of this detection probe was dependent on the binding of the biotinylated Abs with streptavidin conjugated AuNPs [74]. The biosensor utilised both monoclonal and polyclonal Abs in the sandwich-based immunoassay, although more significantly, the biotinylated strategy employed produces randomly orientated Abs. Site-specific biotinylation strategies have been shown to improve the Ab binding activity by up to 33% [75]. In fact, the same study demonstrated that site-specific biotinylated Fab Ab fragments have been shown to be up to five times more effective than those that have been randomly orientated. Another study has shown that immobilising Abs through their carbohydrate moiety produces promising results, [76] as the carbohydrate functional groups are located in the Fc region of an Ab and away from the antigen binding sites. However, this approach cannot be considered when using Ab fragments lacking an Fc region.

An appealing ultimate target of any POCT platform is to provide the capability to effectively detect biomarkers directly in serum. This objective is often restricted and measurements can be distorted due to the natural occurrence of numerous interfering biomolecules present in human serum [77,78]. Several immobilisation approaches have been proposed to assist in mitigating the negative effects of these interfering molecules, some of which incorporate the use of self-assembled monolayers (SAMs), hydrogels and zwitterionic compounds among others [79,80,81,82,83]. Dhawan et al. have designed peptidylated surfaces to minimise biological fouling during cTnI detection [84]. This work incorporated the evaluation of triazolic and non-triazolic peptides to determine the most effective anti-fouling immobilisation surface. The captured Abs were immobilised onto the peptidylated surface with the non-triazolic peptides providing the most promising results. The matrix effects in 10% serum were insignificant as a result of the non-triazolic peptidylated surface. Another group reported on the development of a reduced graphene oxide-based electrochemical aptasensor for the detection of cTnI in which poly(ethylene glycol) modified pyrene (py-PEG) and 1-pyrenecarboxylic acid (py-COOH) were utilised to minimise the interferences of the serum matrix. To optimise the anti-fouling capabilities of the aptasensor, analysis was performed using varying ratios of py-PEG/py-COOH, culminating in the implementation of 20:1 ratio of py-PEG/py-COOH. This resulted in the fabrication of a highly sensitive aptasensor able to detect cTnI down to 1 ng/L and without being significantly affected by endogenous substances present in serum samples that were obtained from patients reporting chest pain. Several examples of assay formats and the associated immobilization strategies employed are given in Table 1.

## 5. Incorporation of Nanomaterials

**Figure 2:**
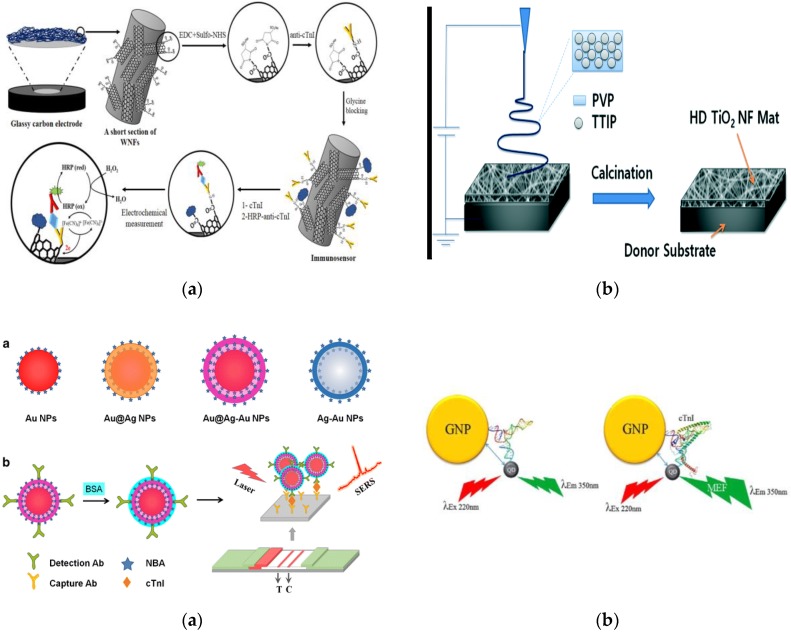
Using nanomaterials in cTnI sensor fabrication [88,92,93,96] (**a**) Electrode fabrication using carbon nanotube-whiskered nanofibers and the bioreceptor conjugation strategy; (**b**) Fabrication process of a nanofiber mat using electrospinning; (**c**) SERS nanoparticles for signal enhancement in a lateral flow assay; (**d**) Luminescence aptasensor detection mechanism showing the reduction in the distance between AuNP and QD enhancing the fluorescent signal (All figures have been re-printed with permission from the publisher).

Nanomaterials are rapidly emerging as key components in the construction of sensor platforms and can be used to achieve outstanding sensitivities and contribute greatly towards the miniaturisation of sensors. They are class of materials distinguished by their size which typically ranges from 1–100 nm and exhibit properties that differ to bulk material [90,91]. The increasingly frequent adoption of nanomaterials is evident within several of the most recent assays and biosensors targeting cTnI, with a wide range of traditional nanomaterials being employed within these detection techniques. Nanotubes made from carbon are composed of either a single layer (single-walled carbon nanotube [SWCNT]) or multiple layers (multi-walled carbon nanotube [MWCNT]). Metals such as gold and silver are used in the synthesis of metallic nanoparticles, “highly branched polymer systems” known as dendrimers are typically less than 10 nm in size, and quantum dots (QDs) which are semiconductor-based nanocrystals display desirable optical properties beneficial in fluorescence detection systems among others [90,92]. Carboxylated MWCNTs are used as the foundation to create whiskered nanofibers by electrospinning, which facilitates the immobilisation of capture Abs [93]. In this work, shown in Figure 2a, the combination of nanofibers and carbon nanotubes aims to provide additional advantages over using one sole form of nanostructure. Noticeably, this study revolves around an electrospinning process which sees the deposition of the nanofibers directly onto the glassy carbon electrode (GCE) surface. Electrospinning is a common fabrication technique that is widely used in the construction of nanofiber-based biosensors. Using electrospinning, titanium dioxide (TiO_2_) nanofibers were deposited onto a silicon wafer to form a TiO_2_ nanofiber mat on which capture Abs could then be immobilised [94]. This particular approach, which is illustrated in Figure 2b, involves the transfer of the nanofiber mat using polydimethylsiloxane (PDMS) offering a unique solution to avoid damaging the nanofiber mat due to its brittle composition and was capable of detecting cTnI at concentrations as low as 4.4 ng/L.

The broad diversity of nanoparticles and the natural properties that they exhibit enables them to fulfil a number of different requirements often associated with biosensors. One research group used zinc oxide nanoparticles as the basis to construct a thin film in the design of an interdigitated electrode biosensor [95]. In this work, the authors outline the fabrication of the interdigitated electrodes, which had been achieved through the use of a conventional photolithography method. Furthermore, the group disclosed the operation of the sensor by explaining that the addition of cTnI onto the electrodes surface, linearly increases the current flow due to the positive charge associated with cTnI represented by its isoelectric point and the negative charge within the zinc nanoparticle-based thin film. A separate group have developed an immunoassay which relies upon surface plasmon resonance (SPR) as the detection method [96]. This work involved synthesising hollow AuNPs to enhance the SPR signal, thus improving the sensitivity of the assay. Bai et al. have gone one step further and synthesised three different types of nanoparticles for surface-enhanced Raman scattering (SERS) detection [97]. Along with using standard AuNPs, the authors have assessed the analytical performance of the lateral flow assay for each nanoparticle individually, all of which are displayed in Figure 2c. Raman spectroscopy is a technique that relies on the inelastic scattering of light consequently occurring due to molecule interaction. The use of metals to amplify these signals is the foundation of SERS and furthermore it has been shown that using nanoparticles with a core and shell structure can further increase the sensitivity [98,99]. The authors have demonstrated that the Au@Ag-Au nanoparticles, which represent a ‘rattle-like’ gold core in a shell composed of a silver coating with a subsequent outer gold coating, exhibited the greatest sensitivities of the nanoparticles that were tested. Interestingly, AuNPs alone had better sensitivity than the other two core–shell-based particles.

Nanoparticles have the distinct advantage over other nanostructures of being finely tuned through altering their shape and particle dimensions [91]. AuNPs are probably the most extensively used nanoparticles, with ideal sensing properties helping boost sensor performance with high stability, outstanding electrical conductivity and prime optoelectronic properties [100,101]. AuNPs and QDs are used as the plasmonic nanoparticles and excitonic semiconducting nanocrystals, respectively, in a metal-enhanced fluorescence detection system [88]. Oligonucleotides were used to connect the AuNPs with the QDs and aptamers to create labelled biorecognition elements. As the cTnI binds to the aptamer, a change occurs in the structure of the linking oligonucleotides, which in turn reduces the distance between the QDs and the AuNP which can be observed in Figure 2d. Hence, the energy transfer between the QDs and the AuNP increases and the fluorescent signal is subsequently enhanced. A feature of this technique which could make it a promising candidate for POC integration is that it is aimed at detecting cTnI in saliva, unlike many of the techniques reviewed which ultimately intend to use blood as the testing sample. Saliva cTnI levels have been shown to correlate with serum levels, although the detectable concentrations are much less than what can be attained in serum samples [102]. Moreover, some cardiac biomarkers do not demonstrate a correlation between serum levels and that detectable in saliva, hence detecting cardiac biomarkers in saliva requires careful consideration [103]. AuNPs and QDs can be chemically mixed together to form a AuNP/QD nanocomposite which exhibits excellent photoelectrochemical properties [104]. Thus, in response to an excitation light source, a photocurrent will be produced corresponding to the AuNP/QD hybrid concentration. This particular immunosensor has a LOD of 1.76 ng/L and will respond to the increase of antigen binding through a reduction in the photocurrent signal. This occurs as the capture Abs are directly immobilised onto the hybrid material and the increase in analyte concentration blocks the excitation light from interacting with the material, resulting in the weaker photocurrent being generated.

Much of the work associated with sensor platforms focuses on synthesising the nanomaterials and effectively generating a stable surface for Ab immobilisation. Zang et al. have aimed to improve ECL luminophore efficiency through the incorporation of zeolitic imidazolate framework nanocrystals, a subclass of metal organic frameworks (MOFs) [105]. MOFs are currently a hot topic in research due to their unique microporous structure, high surface area and tuneable chemical and physical properties, leading to extensive research in energy and gas storage, environmental applications and sensor development [106,107,108,109]. This work involved synthesising a zeolitic imidazolate framework graphene QD nanocomposite, in addition to synthesising a covering AuNP layer to overcome conductivity issues. The authors also electrodeposited AuNPs onto the GCE surface, thus increasing the surface area for available Ab binding sites. Another class of nanomaterials are dendrimers which are radially symmetric tree-like structures consisting of a central molecule or linear polymer core, displaying attractive properties regarding the optimisation of Ab conjugation [110]. The most widely used type of dendrimer is the polyamidoamine (PAMAM) dendrimer which is commonly available with an alkyl-diamine core and tertiary amine branches, although suppliers do offer a variety of functional groups on the branches for a range of Ab immobilisation requirements. These nanostructures contribute greatly towards achieving a picogram-range limit of detection for cTnI [111]. Here, the PAMAM dendrimers were bound to graphene QDs on a gold working electrode, increasing available Ab binding sites and providing specific orientation through the formation of an amide bond, thus enhancing the sensitivity of the electrochemical biosensor. Another group have also used PAMAM dendrimers in the fabrication of a biosensor, once more utilising the amine functional groups for Ab binding [112]. This work is a fitting example of the complex fabrication process involved in constructing some biosensors, with this particular approach based on the development of a three-dimensional nano network incorporating several different nanoparticles and relying on the hybridisation of DNA for the formation of the detection probe.

## 6. Evaluating cTnI Detection Techniques

**Figure 3:**
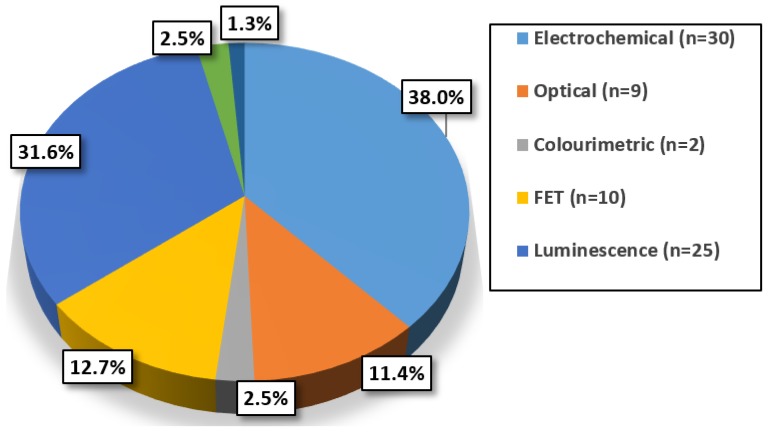
Means of detection employed by researchers for cTnI since 2015. Data was collected for cTnI detection from 77 journal papers reviewed and each technique was appropriately classified based on the assay and biosensor detection mechanism utilised. Two papers have presented two different techniques, one of these being a qualitative colourimetric lateral flow assay.

Assessing the composition and characteristics of the reviewed techniques can assist in identifying trends relating to the most common approaches undertaken and the inherent suitability towards POC integration of these techniques. Certain performance characteristics for each sensor and assay were recorded with particular attention paid towards some of the most relevant criteria in terms of POC integration. Furthermore, as these detection techniques have not been clinically trialed, the precision of each has not been disclosed, hence for the purpose of this review this aspect will not be considered. It is evident from Figure 3 that electrochemical detection methods are the most often used for emerging cTnI assays and biosensors. These typically involve the use of GCEs or screen-printed electrodes (SPEs) and the implementation of an electrochemical technique such as EIS, cyclic voltammetry (CV) or differential pulse voltammetry (DPV). Electrochemical detection is an appealing option, particularly for POCT, as it is easily miniaturised, often is label-free and requires less costly instrumentation than other approaches [113,114].

Kumar et al. have constructed an electrochemical-based immunosensor using CV as the detection method [115]. Nanostructured zirconia was synthesised and electrophoretically deposited onto an indium tin oxide (ITO) coated glass electrode to improve the electron transfer rate and in turn facilitate the detection of cTnI concentrations as low as 100 ng/L. EIS is regularly implemented in cTnI detection and was shown to provide quite high sensitivities as demonstrated in the development of a three-dimensional graphene-MWCNT immunosensor [116]. Measuring the presence of cTnI at concentrations below 1 ng/L is achievable with this biosensor and due to it being label-free, the sensor response time was minimised and the overall fabrication cost reduced.

Over a quarter of the papers reviewed employed a luminescence detection method, the vast majority of which opted for fluorescence or ECL detection. However, there are exceptions, most notably the development of an immunoassay which relies on the emission of visible light from upconversion luminescence (UCL) to detect cTnI [117]. UCL has the distinct advantage of a reduced auto-fluorescence background signal in comparison to some other luminescence methods, in addition to utilising nanoparticles that absorb near-infrared light and emit visible or UV light [118]. The absorbance and emission wavelengths of the lanthanide doped-upconverting nanoparticles used in this assay are 980 nm and 540 nm, respectively. The authors have identified the increased signal-to-noise ratio associated with UCL and aimed to improve upon it by coating the UCNPs with poly(acrylic acid) to reduce non-specific binding of the antibody–UCNP complex onto the solid support. The introduction of free poly(acrylic acid) into the assay buffer assisted in achieving a LOD of 0.48 ng/L, which is considered highly sensitive, but in accordance with some of the other aforementioned methods, may not be in the category of ultra-sensitive assays.

**Figure 4:**
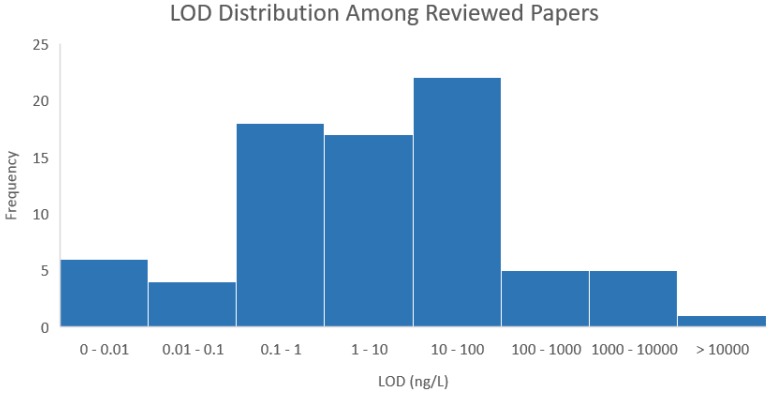
LOD of reviewed papers. Data for the 77 reviewed papers was collected and the assays and biosensors are arranged into subsets based on the LOD. The qualitative colourimetric technique was excluded.

There is a wide LOD range forthe techniques reviewed, with this distribution being depicted in Figure 4. The majority of the detection techniques have been shown to be capable of detecting cTnI below 100 ng/L, with the largest subset, the 10–100 ng/L, demonstrating comparable sensitivities to commercial first generation troponin assays [119,120]. Six of the reviewed methods have a LOD that exceeds 1000 ng/L and it is likely that these techniques would not offer much value in a clinical setting. Additionally, there is no clear correlation between the principle of detection utilized and increased sensitivity as electrochemical, luminescence, FET and optical methods are all represented within the least sensitive subset. 

The time taken between loading the sample containing the biomarker into the instrument or onto the biosensor and receiving the detection results is of paramount interest in comparing the reviewed techniques. This was referred to as the detection method response time and is essential for successful inclusion of any of the most sensitive techniques into a POC platform. Table 2 displays some of the characteristics of the most sensitive techniques arranged in order of response times. It also contains information on immobilisation approaches which are critical to the effectiveness of the techniques. Although it may appear that these are ideal methods for high sensitivity cTnI detection, the detection range of some is not practical. The optical microfiber coupler has a detection range of 8 pg/L and the chemiresistive-based nanobiosensor has a verified detection range of 50 pg/L [71,121]. At the current stage of development, the detection ranges of these biosensors would not be capable of providing significant diagnostic benefit. Additionally, it is evident that many of these high sensitivity techniques incorporate the use of nanomaterials to optimise the Ab immobilisation strategy, once more highlighting the extent to which nanomaterials contribute towards improving sensor sensitivities. Notably, out of the top four response times, three use electrochemical detection. More significantly, the only response times that are less than 20 min are the techniques which do not rely on the use of labels. This is particularly evident in the case of the luminescence methods. Yang et al. have developed an ECL immunosensor that is principally designed around the use of metal–organic frameworks (MOFs) as QD carriers [122]. In this case, the authors have prepared an isorectangular metal–organic framework-3 (IRMOF) from a previously reported method, and encapsulated cadmium telluride QDs (CdTe-QDs) to enhance the ECL intensity. Increasing the density of the immobilised CdTe-QDs is another beneficial consequence of using IRMOFs. The sandwich-type immunosensor format allocates 40 min to allow for cTnI binding and an additional 80 min to accommodate the binding of the IRMOF-based detection probe. An alternative high sensitivity fluorescence-based detection assay capitalises on Single-molecule Array (Simoa) technology [123]. Simoa is based on “the simultaneous counting of singulated capture microbeads” to detect the target analyte [124]. It enables high sensitivity automated detection using standard ELISA reagents and contains a large number of microwells, from which the data can be used to precisely calculate the analyte concentration. This technology is instrumental in the development of an immunoassay for cTnI which has the capacity to detect concentrations as low as 0.01 ng/L. Furthermore, this is a fully automated process potentially suited for POCT. However, the current response time is an element which must be reduced to be seriously considered as POC compatible. The initial stage of the detection process takes 12 min and involves the sample being loaded and the cTnI captured by anti-cTnI-antibody coated paramagnetic beads following the combination of the coated beads with detection Abs. A conjugate of streptavidin-β-galactosidase is introduced during the second step, binding to the biotinylated detection Abs, resulting in labelled cTnI. The overall response time is 45 min, although this system can perform 66 tests per hour, potentially alleviating the dependence on a fast response time to a certain extent.

## 7. Impact of Labels

The previous section has partially introduced the use of labels in assays and biosensors, identifying how they can influence the response time and be used to optimise the sensor performance. Labels are molecules that are “chemically or temporarily” attached to a biorecognition element or antigen, generating a measurable signal representing the analyte concentration [129]. There is a tremendous variety of labels available, offering versatile sensing solutions and range from metallic nanoparticles for electrochemical and optical detection methods to luminescent molecules and enzymes to produce a light emission or colour change. Labelling requires an additional preparation step and, depending on the selected label, suitable storage conditions should be determined. Kim et al. have proposed an enhanced immunogold assay using a silver staining technique for signal amplification [130]. The immunoassay was conducted in a direct and enhanced manner, the latter involving binding of the antigen to AuNP-labelled detection Abs in a sample solution prior to being captured. Following the formation of the assay sandwich complex, and preceding colourimetric measurements, a silver enhancer was introduced which completely covers the AuNPs. Furthermore, the authors successfully demonstrated that the enhanced method detection sensitivity exceeded that of the direct method by two orders of magnitude.

SPR is a technique that is typically conducted without labels, owing to a detection mechanism based on light refraction that is dependent on the difference in the dielectric constant of adjacent materials [131]. However, the adoption of labels can enhance the sensitivity, which Wu et al. have demonstrated [132]. Magnetic-MWCNTs (MMWCNTs) were fabricated by combining Fe_3_O_4_ with MWCNTs, enabling controlled magnetic re-dispersion of the cTnI-conjugated detection probe in a flow cell. The electrode surface was coated with hollow AuNPs to enhance the SPR signal and to facilitate a greater number of binding sites. The aforementioned unlabelled sandwich-type immunoassay, in which Wu et al. relied solely on the completion of the antibody–antigen sandwich complex to enhance the SPR detection sensitivity, was far less sensitive, having a LOD which is 30 times larger than the MMWCNT-based approach [96]. An electrochemical aptamer-based biosensor for cTnI detection was developed using hydrazine labels as electrocatalysts for the reduction of H_2_O_2_ [133]. Here, an aptamer sandwich is formed, using chronoamperometry as the electrochemical detection technique. The response time of this biosensor is 10 min, which is unusually fast for a sensing mechanism that relies on a sandwich format.

**Figure 5:**
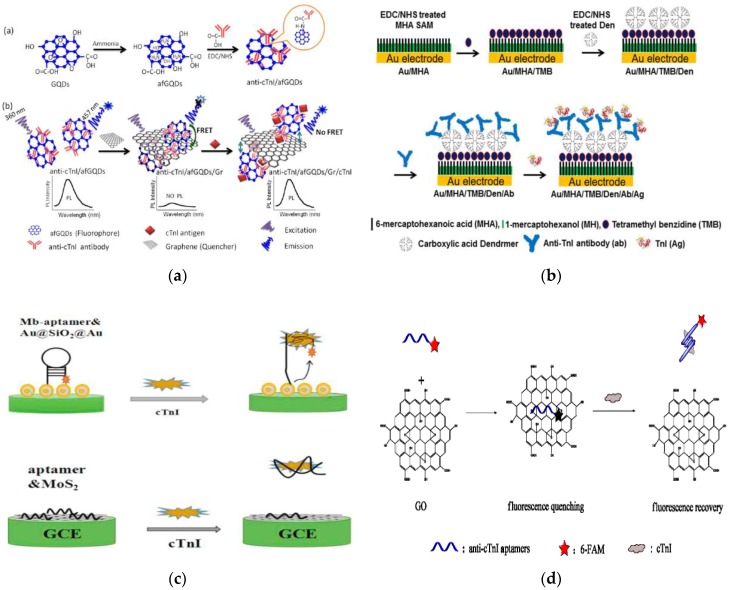
Label-free and novel labelling techniques [134,136,137,138]. (**a**) Detection process scheme for graphene quenching of afGQD fluorophores and subsequent release of Ab upon cTnI binding; (**b**) Fabrication scheme of a ‘label-free’ electrochemical-based biosensor; (**c**) Electrochemical detection technique utilising a core–shell immobilisation surface and alternative ‘label-free’ detection technique utilising MoS_2_ nanosheets for cTnI measurements; (**d**) 6-FAM-modified aptamer dislodged from graphene oxide sheets in the presence of cTnI. (All figures have been re-printed with permission from the publisher).

From compiling the journal papers and investigating the assay formats, it has become evident the effect that labels, or more specifically, the formation of an antibody–antigen sandwich complex has on the detection method response time. The median response time for the techniques which implemented labels was 57.5 min. In contrast, the median response time for assays/sensors constructed without the use of labels was 10 min. Minimising the assay or biosensor response time is key to successful integration into a feasible POC platform, as reducing the turnaround time of a diagnostic test is a crucial aspect on which POCT is based. This fundamental feature of POCT may suggest that some detection methods which inherently involve labels may not be completely suited to POCT without performing parallel analysis. In spite of this, there are inevitably some exceptions with researchers endeavouring to find alternative approaches to standard methods, for example some of the quenching approaches that have been previously discussed. However, those that have already been mentioned had response times of 115 and 35 min, with both of these techniques implementing labels. Bhatnagar et al. described a fluorescence-based biosensor which is illustrated in Figure 5a and has a response time of 10 min [134]. Once again, quenching is essential for the detection mechanism on which this approach is based. The authors use amine-functionalised graphene QDs (afGQDs) to produce the fluorescent emission and also employ the use of graphene (Gr) sheets to quench this signal. The capture Abs are conjugated with afGQDs to form fluorescently-active nanoprobes and are immobilised directly onto Gr sheets. Binding of the cTnI effectively increases the distance of the afGQDs from the Gr sheets, thus reducing the fluorescence quenching effect and recovering some of the fluorescence signal. Hence, as the cTnI concentration is increased, the detectable fluorescence signal will increase accordingly. This approach is capable of detecting cTnI concentrations of 0.192 ng/L and illustrates the adaptability of fluorescence detection techniques and luminescence in general. Furthermore, the authors performed the detection using serum which had previously been centrifuged for 10 min, essentially resulting in an overall response time of 20 min.

The development of a ‘label-free’ photoelectrochemical (PEC) biosensor follows a similar theme, having the capacity to detect cTnI at concentrations as low as 0.3 ng/L [86]. PEC sensors typically use QDs to produce the photocurrent upon absorption of photons. Using QD-labelled detection Abs is a standard approach in the design of a PEC sensor, however, there are numerous alternative sensor configurations [135]. Fan et al. have utilised steric hindrance which capitalises on the binding of the cTnI to restrict the electron transfer between nitrogen and sulphur-doped graphene QDs (N,S-GQDs) and the PBS-ascorbic acid electrolyte. Additionally, this work involved the synthesis of cadmium sulfide (CdS) co-sensitised hierarchical Zn_2_SnO_4_ cubes in which the N,S-GQDs were assembled to optimise the photo-to-current conversion efficiency. In contrast, Akter et al. have presented a novel ‘label-free’ electrochemical biosensor that uses dendrimers as a conjugating junction between 3,3′5,5′–tetramethyldenzidine (TMB) and the capture Abs with the aim of increasing the overall number of immobilised Abs and eliminating the need for an external redox probe [136]. The scheme representing the sensor fabrication is shown in Figure 5b in which the dendrimers had been covalently attached to a TMB-modified 6-mercaptohexanoic acid (MHA) self-assembled monolayer (SAM), with the covalent bonds between the dendrimers and the Abs increasing the stability of the amperometric sensor. The TMB redox couple is attached to the SAM surface and acts as a signal generator and the biosensor can measure cTnI concentrations down to 0.28 ng/L, hence this is a promising approach for POC integration. However, it must be noted that the response time for this immunosensor is 60 min, which may hinder its use in POCT. Another group have developed two different types of assays based on the binding behaviour of different aptamers [137]. Neither method relies on the formation of an antibody–antigen sandwich complex, although the first method which incorporates the use of certain core–shell NPs for generating a suitable immobilisation surface and is illustrated in Figure 5c, conjugates methylene blue to the hairpin ssDNA aptamer. Binding of cTnI altered the structure of the aptamer, consequently increasing the distance of the methylene blue from the electrode surface, thus reducing electron transfer. The second method is based on the use of molybdenum disulphide (MoS_2_) nanosheets as the immobilisation surface. Upon binding of cTnI, the aptamer folded into a rigid tertiary structure and as a result was released from the MoS_2_ nanosheet surface. This had the effect of increasing the electron transfer rate as the aptamer acted as a resisting element, hence the EIS detection technique employed was capable of measuring levels of cTnI as low as 22.8 ng/L. This approach is very similar to the work of another group shown in Figure 5d, in which they have implemented the same concept regarding the dislodgment of the aptamer upon binding with cTnI [138]. Here, the 6-carboxyfluorescein-modified aptamer is immobilised onto a quenching surface consisting of graphene oxide nanosheets. Similarly, the introduction of cTnI causes the aptamer to leave the graphene oxide surface due to there being a high affinity with the antigen, thus reducing the graphene oxide quenching effects and enabling effective detection of cTnI. This technique could detect cTnI as low as 70 ng/L in 25 min, although it cannot be directly compared with the MoS_2_ nanosheet-based method as the response time for that aptasensor has not been disclosed. Other photoelectrochemical methods, unrelated to cTnI detection, have involved nanocrystal-labelled detection Abs for signal amplification through steric hindrance but also by reducing QD photon absorbance and using enzyme-conjugated detection Abs to catalyse hydrolysis reactions [139,140,141].

### Reducing the Response Time

The design of an immunoassay or biosensor will inevitably dictate the response time, with the inclusion of labels heavily influencing this aspect. However, there are several existing methods that can assist in reducing the response time of an assay by enhancing the rate of diffusion [142,143]. Micromixers are devices or structures which are used to improve the rate of diffusion within a solution. They can be easily classified into two categories i.e., active and passive mixers. The active mixers apply external forces to improve the diffusion within a solution whereas passive mixers achieve diffusion through geometric means, typically increasing the contact area and designing restriction elements to induce some degree of turbulence. Certain strategies have been conceived that aim to speed up the detection process, some of which apply to cTnI detection techniques. Lee et al. have implemented AC electrothermal flow into the design of a biosensor in an attempt to overcome the “diffusion limit” [144]. The exertion of electrokinetic forces on the fluid induces a stirring mechanism which affects small molecules, however, the response time for this biosensor remains quite high at 90 min. Another group have developed a SWCNT-based immunosensor consisting of two pairs of electrodes [145]. One pair is used to measure the relative resistance change due to the binding of cTnI and the other set for the dielectrophoretic concentration of cTnI. Using 5 V peak-to-peak signal at 100 kHz, the cTnI was concentrated on the SWCNT, reducing the incubation time from 60 min to 1 min. Furthermore, this immunosensor has a wide linear dynamic range of 1 ng/L to 100 μg/L and a LOD of 0.7 ng/L, illustrating the beneficial outcome of mixer integration on the response time without hindering the sensitivity.

## 8. Transition from Assay to POC Platform

**Figure 6:**
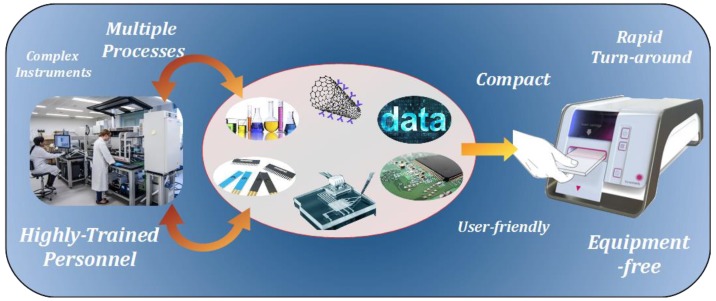
Transitioning from laboratory assays to cartridge-based POC platforms.

Many of the techniques that have already been reviewed encompass only the detection of cTnI, excluding the practical challenges that will face POC integration. POCT must be user-friendly, eliminating the need for highly trained personnel and assisting in promoting the adoption of this technology on a broader sense within healthcare services, particularly in primary care. Fundamental to the design of a POC platform is automation. Loading the sample into the POC device should initiate the sensing procedure and may require mechanical assistance in the form of actuators and also relies on fluid manipulation mechanisms. Furthermore, there are several additional key aspects that link into the engineering design of a POC system, such as sample delivery or cartridge design, electronic circuitry, software development, signal processing and data management. Many of the challenges identified are often encountered during the development of an in vitro diagnostic platform and the specific approach undertaken for each device is highly dependent upon the pre-defined set of design constraints. However, additional demands arise during the development a POC platform due to the need to adhere to recommended POCT criteria. Several of these requirements have been previously mentioned, with other recommendations advocating the use of robust and safe reagents and consumables, that the platform is independent of complex equipment that may be required in sample pre-treatment and the test results are consistent with an established laboratory method [146]. Furthermore, the design of a POC platform will inherently dictate its conformance for large-scale production and the cost effectiveness of the individual test. However, at present, the ambiguous nature of evaluating the economic consequence of POCT compared to laboratory analysis has somewhat restricted its widespread implementation [147]. Figure 6 depicts the transition from a laboratory-based assay to a POC platform, indicating several elements that are often key in the fabrication and operation of a POC device.

Various research groups have attempted to develop an entire sensing device, as seen with the Simoa platform which was previously discussed. Others such as Singh et al. have focused on the development of the microfluidic system in conjunction with the detection method [148]. Here, the authors have fabricated a microfluidic biochip and have nanoengineered a microporous manganese-reduced graphene oxide (Mn_3_O_4_-RGO) nanocomposite for the purpose of increasing the capture Ab loading capacity. In this example, the microfluidic chip, containing a three-electrode configuration for electrochemical detection, represents a precursor to a POC cartridge. Furthermore, this study identified the combination with reduced graphene oxide sheets as a source of many beneficial characteristics such as higher sensitivity and stability, an abundance of functional groups and an improved electrochemical response [149]. Another group have identified the restricting nature of the instrumentation used for fluorescence detection in relation to POC integration, developing a LED-based UV excitation optical analyser to improve the POC compatibility of fluorescence assays [150]. The authors report comparable sensitivities to commercially available fluorescence-based analysers, in combination with a reduced background signal which contributed to the detection of cTnI at levels as low as 0.22 ng/L. An alternative approach reported by Sarangadharan et al. details the development of an electric double layer gated FET-based biosensing system that has achieved a LOD of 2.62 ng/L from an untreated 2 μL blood sample within 5 min [151]. The authors allude to the potential portability of the system and its sensing capability, identifying the clinical benefits of avoiding whole blood pre-treatment as a critical characteristic and highlighting the potential POC compatibility of FET-based biosensors. A separate study details the construction of a time-resolved fluorescence cTnI immunosensing platform which uses europium chelate-encapsulated silica nanoparticles to transfer the excitation energy to lanthanide ions [152]. Kim et al. have identified quenching issues with commercially available lanthanide ion–chelate complexes in water. The authors have constructed a time-resolved fluorescence-based sensing platform, including optical components and a custom power supply to detect cTnI, thus utilising the lanthanide luminophore-encapsulated nanoparticles to overcome the signal quenching and achieve overall greater sensitivities. The construction of POC platforms is a substantial advancement from performing a routine laboratory assay, involving the extraction of the individual elements of an assay and packaging them into a single automated platform. Laboratory analysis often requires conducting several assays in parallel using a single sample for multi-marker measurements. Hence, to effectively optimise the diagnostic efficacy of a POCT platform, introducing multiple marker detection is of paramount interest.

## 9. Multiplexing

Providing an effective diagnosis on the basis of single biomarker detection can prove to be quite tedious, hence many commercial systems offer multiplexing capabilities. Multiplexing incorporates the detection of multiple biomarkers from a single sample, enabling greater confidence in a particular diagnosis. Additionally, multiplexed detection of cardiac markers has been shown to dramatically improve the predictive capabilities of a clinical assay [153,154]. The most recent hs-cTnI assays have reduced the troponin blind period to an hour, however, obtaining an immediate diagnosis for a patient presenting at the ED is critical when dealing with CVD [7]. Multiple reports allude to the negative predictive value of myoglobin when a patient is first presented at the ED and highlight the ineffectiveness of cTnI and cTnT in comparison to myoglobin and CK-MB in diagnosing AMI within the first 2 h [155,156]. Several studies have demonstrated the beneficial outcome of targeting multiple key biomarkers that reflect cardiomyocyte damage. Zethelius et al. have evaluated the prognostic capacity for older men when measuring up to four different cardiac biomarkers, including cTnI [157]. This report has shown that the predictive risk of death due to cardiovascular causes is up to 10 times greater when taking into account the four biomarkers than when only measuring cTnI. Another group have implemented a similar approach, measuring four different cardiac biomarkers and evaluating the diagnostic capability achievable with this method [158]. The study found that there were clear differences in the diagnostic outcome of the method depending on gender and age, with the highest sensitivities being obtained for elderly and female patients. The trend of developing multiplexed detection techniques continues to expand with many reported examples throughout recent cardiac-related literature reports. Shanmugam et al. presented a multiplexed electrochemical immunoassay for the detection of cTnI and cTnT comprised of two sensor arrays on which the authors selectively grew zinc oxide nanostructures onto the working electrodes [159]. Using both EIS and Mott-Schottky electrochemical techniques, this approach could detect both troponins (I and T) at concentrations as low as 1 ng/L. Another group have included cTnI and cTnT in a multiplex detection approach and have also opted to measure the N-terminal prohormone of brain natriuretic peptide (NT-proBNP) citing its diagnostic and prognostic relevance in AMI [160,161]. NT-proBNP is released in greater concentrations from the cardiac ventricles during cardiac stress and has a half-life six times that of BNP. It will naturally be secreted during a cardiac event and can be more easily detected than BNP [162]. This detection technique has integrated 9G DNACHIP technology, a specific DNA microarray technique that uses an oligonucleotide with nine consecutive guanines on AMCA-1,3-dialdehyde (AMCA) slides for specific orientation of the biorecognition element [163]. Here, the authors have followed the DNA-directed immobilisation (DDI) method and conjugated complementary DNA (cDNA) to the capture Abs [164]. The hybridisation between the cDNA enable orientated immobilisation of the capture Abs, thus increasing the binding capacity of the Ab layer. One drawback of this particular method is the long response time for the biosensor. The microarrays were fluorescently analysed for each biomarker after 4 and 15 h, with an improvement in the sensitivity evident for the longer incubation time. For this approach to be compatible with a POC platform, the biosensor response time must be reduced.

Integrating multiplexed detection into POC platforms is a key objective for effectively managing cardiac-related illnesses and this can be further enhanced by targeting a selected array of validated biomarkers. Establishing a combination of the most useful biomarkers in relation to gaining purposeful information is a complicated task due to the wide range of cardiac biomarkers that have been identified. Moreover, there are conflicting reports in relation to the clinical utility of some cardiac markers regarding the improvement of a patient’s health outcome, for instance the prognostic benefits of C-reactive protein have been disputed [165,166,167,168,169]. Some research groups have proposed the implementation of cardiac biomarker algorithms to add diagnostic and prognostic value to multiplexing POCT in addition to reducing the turnaround time. A report by one group outlines an algorithm incorporating cTnI, myoglobin and CK-MB which was used in an ED on patients suffering from chest pain in an attempt to diagnose AMI [170]. This study has shown that when only using cTnI, a negative predictive value (NPV) of 99.9% was achieved within 3 h from admission to the ED, however the positive predictive value (PPV) was only 36.4%. Measuring the three biomarkers and implementing the devised algorithm resulted in a significant increase of the positive predictive value to 92.4%. A different approach, presented by Jaeger et al., had validated a novel algorithm using hs-cTnI to achieve a one hour rule-in/rule-out of AMI [171]. This work demonstrated that using an algorithm solely based on cTnI generated a NPV of 100% and a PPV of 70% within one hour of admission to the ED. Using the hs-cTnI in this study has assisted in obtaining a much improved PPV, however measuring multiple biomarkers in conjunction with implementing a suitable algorithm offers the most promising diagnostic and prognostic prospects. 

## 10. POC Compatible Techniques

**Figure 7:**
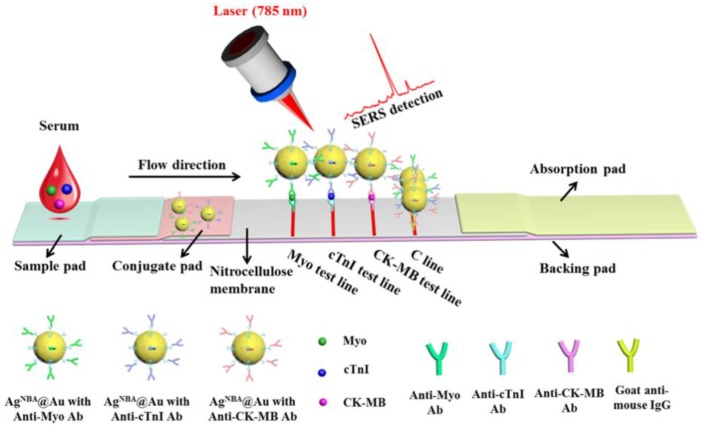
Diagram of a SERS lateral flow assay used to achieve multiplex detection [176] (Figure was re-printed with permission from the publisher).

Throughout the course of this review, it has become apparent that certain detection techniques are inherently more suited towards POC integration than others. Label-free techniques have the advantage of faster response times as there are fewer stages in the detection process. Additionally, the biosensor fabrication costs should be reduced, leading to more affordable POC platforms. Electrochemical and FET-based biosensors are the most easily miniaturised, being highly suited to portable POC devices. However, devising a strategy for interfacing automated microfluidic manipulation for these can prove to be difficult. Ultimately, the route that is taken in the design of POC platforms is down to preference; nonetheless, the settings in which these tests are conducted can have a substantial impact on the platform design. Lateral flow assays are often more suited to low-resource settings as a consequence of their basic fabrication needs. There are numerous advantages of using lateral flow assays as they can be fabricated at a low cost, can easily be mass produced, provide a simple and versatile solution, typically do not require pre-treatment and often offer short response times [172,173]. One group have developed core–shell-structured NPs loaded with Nile-red fluorescent dye which are utilised in a cTnI lateral flow assay [174]. The Nile-red fluorescent dye had been specifically selected to reduce the interferential autofluorescence background signal generated by biomolecules in plasma. This assay enabled human plasma to be directly applied to the sample pad and the subsequent detection process took 15 min. Additionally, the lateral flow test strip was stable in storage for up to 3 months, as well as obtaining measurements of cTnI down to 16 ng/L and thus emerging as a prime candidate for POC integration. An alternative fluorescence-based lateral flow assay incorporated streptococcal protein G (SPG) on the surface of microspheres to optimise Ab orientation [175]. The Ab orientation was based on specific binding of the Fc region with the SPG through chemical bonds utilising EDC. This lateral flow assay had a response time of 15 min and is highly sensitive, detecting cTnI levels as low as 32 ng/L. Zhang et al. have developed a multiplexed lateral flow assay using SERS as the detection technique and similar to many SERS techniques, the authors have used bimetallic particles to enhance the assay sensitivity [176]. Illustrated in Figure 7, this design has three separate test lines for each of the cardiac biomarkers with a response time of 15 min and no requirement for sample pre-treatment. It is a sandwich-type immunoassay, which has bimetallic nanoparticle conjugated detection Abs attached to the conjugate pad and is also highly sensitive, with a cTnI LOD of 0.44 ng/L and comparable LODs for the other two biomarkers.

Although lateral flow assays have been portrayed as a low-cost option, they have similar diagnostic capabilities to other assay configurations when antibody labels are employed. This is evident for some of the commercially available cardiac lateral flow assays with several utilising fluorophore labels to provide quantitative measurements, although the majority of commercial lateral flow assay-based POC devices are qualitative [177,178]. Examining the instrumentation required to conduct the above assay suggests that it may not be possible to even construct a relatively small benchtop POC platform due to the Raman microscope system required. However, there is an increasing amount of work being focused on the development of miniaturised instrumentation from SERS microscopes to portable SPR platforms and mini potentiostats for electrochemical sensors [179,180,181,182,183]. Concisely categorising a detection technique as POC compatible can be difficult due to many aspects that must be taken into account. However, lateral flow assays inherently display many attributes which support the claim that they are ideal for POC integration. Furthermore, optimising the design of lateral flow assays to enable widespread highly sensitive detection of cTnI and other cardiac biomarkers is a major target which should be actively pursued. 

## 11. Conclusions

Improving the sensitivity of cTnI assays and cTnI POC platforms provides added benefit to clinicians by enabling a more rapid implementation of a suitable management plan to ultimately lead to a better health outcome for the patient. POC platforms in particular have the potential to reduce pressure on emergency departments (EDs) and enable the widespread distribution of affordable portable cardiac diagnostics. Currently, the analytical performance of cTnI POC platforms trails that of the established laboratory cTnI assays, underpinning the need to further develop POCT technology. Additionally, accurately detecting cTnI is a challenging task due to the variation in the forms of the circulating cTn complex, the concentration fluctuation between differing gender and age groups and the further complications arising from the many factors that impinge on binding with the biorecognition element, inducing skewed concentration measurements and generating false positives and potentially false negatives. Biorecognition element selection is a key consideration that significantly contributes towards the influence of the interfering factors. Utilising antibodies against multiple epitopes, including those at the C- and N-termini of troponins and employing the use of fragment antibodies is shown to enhance accurate and appropriate antigen recognition and reduce non-specific interactions emanating from the serum matrix. Similarly, the implementation of a suitable immobilisation strategy can further assist in improving anti-fouling measures, with multiple techniques having been outlined.

Emerging assays and biosensors have the capacity to detect cTnI at increasingly reduced concentrations and the introduction of nanomaterials has greatly assisted in the development of detection techniques that offer ultra-sensitive performances. Furthermore, the diversity of nanomaterials facilitates the development of novel detection techniques and can help to overcome current deficiencies apparent in some traditional detection methods, minimising the background fluorescence signal using upconverting NPs, influencing the fabrication of ‘label-free’ techniques and accommodating the production of POC compatible quantitative lateral flow assays. The optimisation of cardiac diagnostic POC platforms requires the adoption of an effective multiplexed panel in conjunction with suitable biomarker algorithms to enable the most complete diagnosis in addition to providing increased predictive value long-term and at time of initial presentation to the ED. Targeting the introduction of a disease-specific standardised panel of biomarkers will ultimately provide clinicians with enhanced diagnostic capabilities and will increase the efficacy of cardiac diagnostics, although at an initial increase in cost due to the development and optimisation of the multi-marker panel for POCT. Finally, the combination of efficient anti-fouling measures, the utilisation of compatible detection methods, and sufficient consideration of the intended test setting will ensure the delivery of the most effective POC platform.

## 12. Reprinting Figures

Figure 1 Reprinted from *Sensors and Actuators* A: Physical, 259, Adzhri R., M.K. Md Arshad, Subash C.B. Gopinath, Ruslinda A.R., M.F.M. Fathil, C. Ibau, M. Nuzaihan M.N., Enhanced sensitivity mediated ambipolar conduction with p-type TiO_2_ anatase transducer for biomarker capturing, 57–67, 2017, with permission from Elsevier.

Figure 2a Reprinted from *Talanta*, 182, Babak Rezaei, Ahmad Mousavi Shoushtari, Mohammad Rabiee, Lokman Uzun, Wing Cheung Mak, Anthony P.F. Turner, An electrochemical immunosensor for cardiac Troponin I using electrospun carboxylated multi-walled carbon nanotube-whiskered nanofibres, 178–186, 2018, with permission from Elsevier. 

Figure 2b Obtained from an open-source journal.

Figure 2c Reprinted with permission from Springer Nature, *Analytical and Bioanalytical Chemistry*, Functionalized Au@Ag-Au nanoparticles as an optical and SERS dual probe for lateral flow sensing, Tingting Bai, Meng Wang, Min Cao et al., 2018.

Figure 2d Reprinted from *Engineering in Life Sciences*, 17, Zahra Rezaei, Bijan Ranjbar, Ultra-sensitive, rapid gold nanoparticle-quantum dot plexcitonic self-assembled aptamer-based nanobiosensor for the detection of human cardiac troponin I, 2016, with permission from John Wiley and Sons.

Figure 3 and Figure 4: [37,38,48,49,50,57,58,61,65,66,68,69,70,71,74,84,85,86,87,88,89,93,94,95,96,97,104,105,111,112,115,116,117,120,121,122,123,125,126,127,128,130,132,133,134,136,138,144,145,148,150,151,159,161,174,175,176,184,185,186,187,188,189,190,191,192,193,194,195,196,197,198,199,200,201,202].

Figure 5a Reprinted from *Biosensors and Bioelectronics*, 79, Deepika Bhatnagar, Vanish Kumar, Ashok Kumar, Inderpreet Kaur, Graphene quantum dots FRET based sensor for early detection of heart attack in human, 495–499, 2016, with permission from Elsevier.

Figure 5b Reprinted from *Biosensors and Bioelectronics*, 91, Rashida Akter, Bongjin Jeong, Yong-Mi Lee, Jong-Soon Choi, Md. Aminur Rahman, Femtomolar detection of cardiac troponin I using a novel label-free and reagent-free dendrimer enhanced impedimetric immunosensor, 637–643, 2017, with permission from Elsevier.

Figure 5c Reprinted from *Biosensors and Bioelectronics*, 113, Xiujuan Qiao, Kunxia Li, Jinqiong Xu, Ni Cheng, Qinglin Sheng, Wei Cao, Tianli Yue, Jianbin Zheng, Novel electrochemical sensing platform for ultrasensitive detection of cardiac troponin I based on aptamer-MoS2 nanoconjugates, 142–147, 2018, with permission from Elsevier.

Figure 5d Reprinted with permission from Springer Nature, *Analytical and Bioanalytical Chemistry*, A novel fluorescent aptasensor for the highly sensitive and selective detection of cardiac troponin I based on a graphene oxide platform, Dongkui Liu, Xing Lu, Yiwen Yang et al., 2018.

Figure 7 Reprinted from *Biosensors and Bioelectronics*, 106, Di Zhang, Li Huang, Bing Liu, Haibin Ni, Liangdong Sun, Enben Su, Hongyuan Chen, Zhongze Gu, Xiangwei Zhao, Quantitative and ultrasensitive detection of multiplex cardiac biomarkers in lateral flow assay with core–shell SERS nanotags, 204–211, 2018, with permission from Elsevier.

## Figures and Tables

**Table 1:** Examples of assay components and immobilisation strategies for cTnI detection.

Detection Approach	Biorecognition Element	Immobilisation Strategy	LOD
Optical [85]	Monoclonal Antibody	Ab/EDC/NHS/Dextran SAM/Tboc/Si Wafer	5 ng/L
Photoelectrochemical [86]	Antibody *	Ab/EDC/NHS/TGA/CdS/N,S-GQDs/Zn_2_SnO_4_/ITO	0.3 ng/L
Luminescence [87]	Monoclonal Antibody	Ab/Ru/EDC/NHS/PAMAM/AuNPs/Nafion/GCE	12 pg/L
Electrochemical [61]	MIPs	MIPs/MWCNT/GS/GCE	0.8 ng/L
Luminescence [88]	Aptamer	QD/EDC/NHS/L1/Aptamer/L2/DTT/AuNP	7.2 μg/L
Luminescence [89]	Antibody*	Biotin-Ab/Streptavidin/Magnetic-NPs/GCP	0.2 ng/L

* Type of antibody has not been disclosed. Tboc—10-N-Boc-Amino-dec-1-ene; Si Wafer—Silicon wafer; TGA—Thioglycolic acid; CdS—Cadimium sulphur; N,S-GQDs—Graphene quantum dots doped with nitrogen and sulfu; Zn_2_SnO_4_—Zinc stannate; ITO—Indium tine oxide; Ru—Tris (4,4_-dicarboxylicacid-2,2_-bipyridyl) ruthe-nium(II) dichloride; GCE—Glassy Carbon Electrode; GS—Graphene Nanoplatlets; QD—Quantum Dot; L1—Oligonucleotide linker (GGTGGTGGT- C6 Amine); L2—Oligonucleotide linker (Thiol-GAAGAAGAA); DTT—Dithiothreitol; GCP—Glassy carbon plate.

**Table 2:** Comparison of ultra-sensitive cTnI sensors and assays.

Detection Method	Immobilisation Approach	Response Time (min)	LOD (ng/L)	Labels
Chemiresistance [121]	Ab/EDC/NHS/SU-8/MWCNT	1	0.02	None
Cyclic Voltammetry [111]	Ab/PAMAM/GQD/ATP/Au/SPE	10	0.025	None
Optical Microfiber Coupler [71]	Ab/EDC/NHS/PAA/PDDA/OMC	10	0.002	None
EIS & Mott-Schottky [125]	Ab/AUPA/ZnO/Au & Ab/DSP/ZnO/Au	15	0.1	None
Fluorescence [123]	Ab/Paramagnetic Beads	45	0.01	Biotinylated detection Ab/SβG
ECL [87]	Ru/EDC/NHS/PAMAM/AuNPs/Nafion/GCE	115	0.012	Detection Ab/PtCu_3_ NC/FA/GOD
ECL [122]	Ab/Au@TiO_2_/GCE	120	0.00046	Detection Ab/EDC/NHS/CdTe@IRMOF-3@CdTe
DPV—Anodic Stripping Voltammetry [126]	Ab/ABA/MWCNTs/GCE	240	0.0011	CdS/Detection Ab/ALP/AuNF
ECL [127]	Ab/TEOA@AuNP/GCE	-	0.0055	RuSiO_2_
Total Internal Reflection [128]	Ab/Protein A/G/DSP/Au/Cr	-	0.0000144	Detection Ab/AgNP

DPV—Differential pulse voltammetry; ATP—4 aminothiophenol; SPE—Screen-printed Electrode; PAA—poly-(acrylic acid); PDDA—Poly-(diallyldimethylammonium chloride); OMC—Optical Microfiber Couple; AUPA—aminoundecyl phosphonic acid; ZnO—Zinc Oxide; DSP—dithiobis(succinimidyl propionate); GCE—Glassy Carbon Electrode; Au@TiO2—Gold nanoparticle modified titanium oxide; ABA—3-aminophenylboronic acid; TEOA@AuNP—triethanolamine-modified gold nanoparticles; Au/Cr—Gold-chromium; SβG—streptavidin-β-galactosidase; PtCu3 NC—PtCu3 alloy nanocrystals; FA—Folic Acid; GOD—Glucose oxidase; CdTe@IRMOF-3@CdTe—Isorectangular metal organic framework accelerator enriched cadmium telluride quantum dots; CdS—Cadmium sulphide quantum dots; —Alkaline phosphatase.
